# Identifying Patient Access Barriers for Tumor Necrosis Factor Alpha Inhibitor Treatments in Rheumatoid Arthritis in Five Central Eastern European Countries

**DOI:** 10.3389/fphar.2020.00845

**Published:** 2020-06-05

**Authors:** András Inotai, Dominik Tomek, Maciej Niewada, László Lorenzovici, Martin Kolek, Jakub Weber, Anne-Katrin Kurrat, Emese Virág Kiss, Zoltán Kaló

**Affiliations:** ^1^Syreon Research Institute, Budapest, Hungary; ^2^Center for Health Technology Assessment, Semmelweis University, Budapest, Hungary; ^3^Faculty of Medicine, Slovak Medical University in Bratislava, Bratislava, Slovakia; ^4^Department of Experimental and Clinical Pharmacology, Medical University of Warsaw, Warsaw, Poland; ^5^HealthQuest Sp. zoo Sp. k., Warsaw, Poland; ^6^Faculty of Technical and Human Sciences, Sapientia University, Tirgu Mures, Romania; ^7^Syreon Research Romania, Tirgu Mures, Romania; ^8^OAKS Consulting s.r.o., Prague, Czechia; ^9^Erasmus School of Health Policy and Management, Erasmus University of Rotterdam, Rotterdam, Netherlands; ^10^National Institute of Rheumatology and Physiotherapy, Budapest, Hungary

**Keywords:** rheumatoid arthritis, biologic, Central Eastern Europe, access barriers, TNF alpha inhibitor, pharmaceutical policy, patient access

## Abstract

**Introduction:**

Although there is a significant utilization gap of biologic medicines in the EU, many studies estimate equity in patient access to biopharmaceuticals only based on their availability on the national list of reimbursed medicines. Hidden access barriers may facilitate financial sustainability of pharmaceuticals in less affluent EU countries; however, they have rarely been documented in scientific publications. Our objective was to explore these access barriers for tumor necrosis factor (TNF) alpha inhibitors in rheumatoid arthritis (RA) in five Central and Eastern European countries.

**Methods:**

A detailed interview guide was developed based on multi-stakeholder workshops and a targeted literature review. In each participant country 3-3-3-3 interviews with payers, rheumatologists, patients/patient representatives, and industry representatives were conducted. Responses were aggregated at a country level and validated by primary investigators in each country.

**Results:**

Limited number of RA centers and consequently significant travelling time and cost for patients in distant geographical areas, uneven budget allocation among centers, limited capacity of nurses, narrowed patient population in national financial protocols compared to international clinical guidelines in initiating or continuing biologics, high administrative burden in prescribing biologics and limited health literacy of patients were the most relevant barriers to timely patient access in at least three participant countries.

**Conclusion:**

Assessing only the availability of TNF alpha inhibitors on the national list of reimbursed medicines provides limited information about real-world patient access to these medicines. Revealing hidden access barriers may contribute to initiate policy actions which could reduce inequity in patient access.

## Introduction

Financing high cost biologic pharmaceuticals represent supply side pressure on health expenditure in less affluent European countries. However, external price referencing and parallel trade of pharmaceuticals provide limited room for differential list pricing of patented medicines in Europe (Kanavos and Vandoros, 2011; Kaló et al., 2013; Elek et al., 2017). Due to lower willingness to pay for one unit of health gain and lower overall healthcare spending in certain Central Eastern European (CEE) countries, biologic medicines are often not cost-effective in the CEE region at launch price levels of higher-income countries (Kaló et al., 2012). Despite the more significant demand for modern medicines generated by worse overall health status compared to higher-income European countries (OECD, 2018), public funding of these medicines is associated with a considerable financial burden for the CEE region. Although there is a significant difference in utilization of biologic medicines recognized as ‘standard of care’ within the EU (Kobelt and Kasteng, 2009; Orlewska et al., 2011; Putrik et al., 2014a; Jönsson et al., 2016; Kostic et al., 2017; Baumgart et al., 2019), many studies estimate equity in patient access to pharmaceuticals only based on their availability on the national list of reimbursed medicines (Kawalec et al., 2017; Kamusheva et al., 2018). As an example, the Patients Waiting to Access Innovative Therapies (Patients W.A.I.T.) is a database maintained by the European Federation of Pharmaceutical Industries and Associations (EFPIA), which monitors delays between dates of marketing authorization date and reimbursement (EFPIA, 2019). Hidden access barriers to reimbursed medicines may facilitate financial sustainability of high-cost pharmaceuticals especially in less affluent countries, however, they have been rarely documented in scientific publications (Inotai et al., 2018).

A gap between international clinical guidelines and national guidelines or financial protocols influenced by local budgetary constraints partially explains unequal real-world patient access to reimbursed biologic disease modifying anti-rheumatic drugs (bDMARDs) in rheumatoid arthritis (RA) in different European countries (Kaló et al., 2017). Our objective was to explore and create a taxonomy for access barriers by using the case study of tumor necrosis factor (TNF) alpha inhibitors (a group of active compounds where biosimilars are also available) in RA as an illustrative example based on the experience from 5 CEE countries.

## Materials and Methods

A series of multi-stakeholder workshops were organized in Bratislava (Slovakia) with the participation of more than 30 health policy and health care financing experts, rheumatologists, and patient representatives. Based on guided discussion with these experts a detailed interview guide was drafted in an iterative development process with objective of listing all theoretical access barriers to bDMARDs in RA. Potential access barriers were categorized according to which stakeholder group—including prescribers, patients and manufacturers—is primarily targeted by the policy measure (Inotai et al., 2017). Those barriers influencing prescribers were allocated to additional subcategories depending on whether they are implemented at national level (e.g., restrictive clinical guidelines), institutional level (e.g., restricted drug budget or diagnostic budget for hospitals) or at the level of individual prescribers (e.g., significant incremental administrative burden to prescribe biologics).

As a next step targeted literature review was performed on PubMed to validate the initial list of access barriers. The usage of the search syntax, as described in **Table 1**, resulted in 242 eligible abstracts on 12 February 2019. Title and abstract screening identified 27 potentially relevant full text articles for review. References of full text papers were also reviewed by snowball method, resulting an additional five hits. The 32 potentially relevant full text papers contained 15 articles (Brennan et al., 2004; Kobelt and Kasteng, 2009; Orlewska et al., 2011; Balanescu and Wiland, 2013; Laki et al., 2013; Pavelka et al., 2013; Péntek et al., 2014; Putrik et al., 2014b; Gulacsi et al., 2015; Gulacsi et al., 2017; Kaló et al., 2017; Nikiphorou et al., 2017; Codreanu et al., 2018; Kotulska et al., 2018; Batko et al., 2019) mentioning at least one barrier. Full extraction of these papers to identify potential additional access barriers confirmed the completeness of the draft interview guide.

**Table 1:** Search syntax for the targeted literature review.

Search syntax	Number of hits
(rheumatoid arthritis OR inflammatory arthritis OR polyarthritis OR RA) AND (biologic treatment OR biologic OR biological OR biobetter OR biopharmaceutical OR biodrugs OR tnfa inhibitor OR tnfa blocker OR tnf alfa inhibitor OR tnf alfa blocker OR etanercept OR infliximab OR adalimumab OR certolizumab OR golimumab OR disease modifying anti rheumatic drug OR dmard OR biosimilar OR similar biologic) AND (CEE OR Central Eastern Europe OR Central-Eastern Europe OR Central/Eastern Europe OR Central Europe OR Eastern Europe OR Hungary OR Slovakia OR Romania OR Poland OR Czech) AND (financing protocol OR financing guideline OR financial protocol OR financial guideline OR patient access OR access barrier OR access restriction OR access constraint OR access limitation OR access limit OR price-volume-agreement OR volume limit OR delayed reimbursement OR co-payment OR copayment OR out of pocket OR treatment duration OR treatment cycle OR restricted indication OR limited prescription OR institutional limit OR institutional quota OR hospital quota OR hospital restriction OR institutional restriction OR hospital limit prescriber limit OR prescriber quota OR prescriber restriction OR prescription quota OR prescription limit OR prescription restriction OR second line treatment OR third line treatment OR travel time OR travel cost OR travel burden OR waiting time OR waiting list OR waiting burden OR delay)	242*

*on 12 February 2019.

The final interview guide consisted of 59 potential access barriers with detailed explanations in the abovementioned categories. If a respondent confirmed that a particular barrier to prevent RA patients from timely access to TNF alpha inhibitor medicines existed in his/her own country, the interviewer marked this type of barrier with an ‘X’. Respondents’ personal experiences, explanations or anecdotes provided additional insights in a ‘Comments’ column. If the respondent was unsure about the existence of a potential access barrier, it was considered a ‘no’ response. Finally, although the iterative development during the workshops and the literature review already ensured thematic saturation of the interview guide, respondents had the opportunity to indicate any additional access barriers after each main section that were not listed.

Access barriers were explored in 5 CEE countries, including Czech Republic (CZ), Hungary (HU), Poland (PL), Romania (RO), and Slovakia (SK). Primary investigators in each country validated the local relevance of the interview guide prior to the interviews. In each country anonymous interviews were conducted with representatives of different stakeholder groups including 1) payers, 2) prescriber rheumatologists, 3) patients/patient representatives, and 4) pharmaceutical companies. Based on limited number of experts among payers with sufficient experience related to national coverage and related policy decisions of TNF alpha blockers, and pharmaceutical companies with TNF alpha blockers in their drug portfolio, 3-3-3-3 participants were targeted in each stakeholder group to facilitate the feasibility of our study.

By assuming less prevalent access barriers for patients treated in prominent national institutes, an explicit criterion of interviewing prescribers and patients both from the capital and outside the capital was considered. Besides this general guidance the selection of interview respondents was based on the discretion of the primary investigator. Overall, 3 participants in 4 different stakeholder groups resulting 12 interviews were expected in each of the 5 participant countries.

As patient representatives were also interviewed, the study protocol was approved by the Medical Research Council—Scientific and Ethical Committee in Hungary (number of ethical approval: 10220-2/2019/EKU), and research was performed in accordance with the ethical standards laid down in the 1964 Declaration of Helsinki (WMA, 2013). No sensitive data were collected regarding the patients’ individual health status in the anonymous survey, and according to national legislation patient representatives were asked to sign a written informed consent form.

After conducting the interviews, primary investigators were asked to validate the list of reported barriers and explore the reasons behind the observed inconsistencies, based on their in-depth knowledge of the system in their own country. Finally, access barriers with potential overlap were merged and classified into five categories by researchers, based on their “mechanism of action”: volume control, price control, administrative control, disincentive, and patient illiteracy.

### Validity of Results

Five different steps ensured the validity and comprehensiveness of listed access barriers. (1) Thorough and iterative discussions with multiple stakeholders from several CEE countries helped to compose a detailed list of theoretical access barriers influencing prescribers, patients, and manufacturers in the CEE region. (2) Targeted literature review helped to confirm that no additional access barriers were described in scientific publications. (3) Prior review by primary investigators from each country ensured the interpretability of the items listed in the interview guide. (4) Responders provided positive feedback on the face validity of listed potential access barriers and no additional major access restrictions were mentioned during the interviews. (5) Primary investigators in the five countries—who are also co-authors of this paper—aggregated the interview results and critically appraised and validated the relevance of each reported barrier based on their in-depth country specific knowledge.

## Results

**Table 2** provides an overview on characteristics of responders. Our intention was to select prescribers and patient representatives both from- and outside the capital. In Romania only 2 patient representatives participated in the survey, and one additional prescriber was added to complete the 12 interviews.

**Table 2:** Descriptive characteristics of responders.

	CZ			HU			PL			RO			SK			Total
	All	*From the capital*	*Outside the capital*	All	*From the capital*	*Outside the capital*	All	*From the capital*	*Outside the capital*	All	*From the capital*	*Outside the capital*	All	*From the capital*	*Outside the capital*	
(1) Decision maker/payer	3			3			3			3			3			15
(2) Prescriber/rheumatologist	3	*1*	*2*	3	*1*	*2*	3	*1*	*2*	4	*1*	*3*	3	*2*	*1*	16
(3) Patient representative	3	*1*	*2*	3		*3*	3	*1*	*2*	2	*1*	*1*	3	*1*	*2*	14
(4) Industry representative	3			3			3			3			3			15
* Original manufacturer*	*1*			*2*			*2*			*1*			*2*			*8*
* Biosimilar manufacturer*	*2*			*1*			*1*			*2*			*1*			*7*
**Total**	12			12			12			12			12			60

CZ, Czech Republic; HU, Hungary; PL, Poland; RO, Romania; SK, Slovakia.

**Table 3** depicts the aggregated list of 32 access barriers which were reported in at least one of the participant countries. In each country the list consists of all items that were mentioned by at least one of the respondents and validated by the primary investigator. 14 access barriers were reported from at least three participant countries.

**Table 3:** Patient access barriers to TNF alpha inhibitors in rheumatoid arthritis, mentioned by at least in one interview in a country.

Type of barrier or restriction	Country level restrictions (7)	CZ	HU	PL	RO	SK
Volume control (patient number)	National limit on patient number for reimbursed biologics	X		X		
Volume control (budget)	National budget limit for biologics	X		X		
Administrative control	Some biologic medicines are not reimbursed for all indications	X				
Volume control (companion diagnostics)	National volume limit on diagnostics necessary to initiate or continue biologics				X	
**Administrative control**	**Delays in the update of national reimbursed drug list or implementation of national procurement**	**X**	**X**			**X**
**Administrative control (allocation)**	**Allocation of budget or volume among treatment centers is not reflective to regional disease prevalence**	**X**	**X**	**X**		
Administrative control	Logistic, administrative or communication problems related to the system of ordering biologics for patients	X				X
	Restrictions at regional or institutional level (8)	CZ	HU	PL	RO	SK
**Volume control (patient number)**	**Explicit or implicit institutional limit on patient number for biologics**	**X**	**X**	**X**		
**Administrative control**	**Too limited number of treatment centers with prescribing rights**	**X**	**X**	**X**	**X**	**X**
Volume control (budget)	Institutional budget limit for biologic medicines	X		X		
Administrative control	Regular delays in the implementation of institutional procurement			X		
**Volume control (companion health care service or diagnostics)**	**Institutional volume restriction for outpatient visits or diagnostics (e.g., due to limited budget or capacities)**	**X**	**X**	**X**	**X**	**X**
Administrative control	Prescription of biologics is limited to selected individuals or departments	X				
**Volume control**	**Insufficient human resource capacities to administer intravenous biologics**	**X**	**X**	**X**		**X**
Disincentive	Unattractive (or even negative) institutional margin for prescribing biologics			X		
	Restrictions impacting individual prescribers (4)	CZ	HU	PL	RO	SK
**Volume control** **(patient eligibility)**	**Restrictive local guidelines or financial protocols compared to international guidelines to** **- initiate or continue biologics, or** **- limit treatment duration, or** **- reduce therapeutic dose for patients with remission**		**X**	**X**	**X**	**X**
**Disincentives**	**Prescription practice of biologic medicines is monitored and associated with financial or other disincentives for prescribers above the threshold**		**X**	**X**		**X**
Administrative control	Difficulties in getting the license for prescribing biologic medicines	X				X
**Administrative control**	**Significant administrative burden related to prescribing biologics**	**X**		**X**		**X**
	Restrictions impacting patients (8)	CZ	HU	PL	RO	SK
**Volume control**	**Explicit or implicit waiting list for patients to access biologics**	**X**	**X**			**X**
**Patient illiteracy**	**Restricted referral of patients to treatment centers (due to ignorance or disincentives by primary care); insufficient information about the benefits of biologics for patients**	**X**	**X**			**X**
**Disincentive (indirect costs)**	**In distant geographical areas significant travel time and cost to treatment centers**	**X**	**X**	**X**	**X**	**X**
Disincentive (indirect costs)	Patients have to visit RA or other specialists for treatment monitoring more frequently than administering the next dose		X			
Disincentive (indirect costs)	Patients have to travel to treatment centers frequently (e.g., in every 1-3 month) to renew their prescriptions		X	X		
**Disincentive (indirect costs)**	**Availability of biologics only in selected retail/hospital pharmacies**		**X**	**X**		**X**
Disincentive (direct costs)	Significant co-payment for diagnostic tests				X	
Disincentive (direct costs)	Administration of selected (intravenous) biologics only in hospitals	X		X		
	Restrictions impacting manufacturers (5)	CZ	HU	PL	RO	SK
Administrative control	Extensive requirements (beyond standard Health Technology Assessment) to apply for public reimbursement	X				
Administrative control	In selected cases timelines of pricing and reimbursement decisions compared to transparency directive are not kept	X		X		
Administrative control	Delayed decision on reimbursement applications of new biologics compared to other countries (e.g., not before positive recommendation in X other countries)	X				X
**Price control**	**Strict external price referencing rules prevent or delay market access of new biologics in countries with low price levels**	**X**		**X**		**X**
Volume control	Strict price volume agreement for manufacturers creates self-regulation for manufacturers to control their sales	X				

Barriers reported from at least 3 countries are highlighted with bold letters.

CZ, Czech Republic; HU, Hungary; PL, Poland; RO, Romania; SK, Slovakia.

Prescribers were targeted mainly by administrative and volume control mechanisms. Patient behavior was targeted dominantly by different disincentives. Manufacturers were influenced mostly by administrative tools.

## Discussion

There is a strong political pressure on payers to add new biologic medicines to the list of reimbursed pharmaceuticals. This pressure is coming from multiple stakeholders, including pharmaceutical manufacturers (see the EFPIA W.A.I.T. index), physicians who would like to prescribe new therapies for their patients and patients who would like to have access to new therapies. Finally, politicians also prefer making announcements on the accessibility of innovative drugs especially in politically sensitive periods, such as election campaigns (Ozierański et al., 2012; Robertson et al., 2013; Inotai et al., 2014).

However, in CEE countries inclusion of new pharmaceuticals into the reimbursement list at price levels of more affluent countries may compromise financial sustainability of the pharmaceutical budget. To meet the expectations of different stakeholders, payers in CEE countries add innovative medicines to the reimbursement list with budget control mechanisms in order to collect the political credit of reimbursing new medicines in a way which still ensures the sustainability of health care financing. Nevertheless, from the perspective of patients the majority of budget control mechanisms represent access barriers to reimbursed pharmaceuticals.

As a consequence of these hidden access barriers, the practice of estimating real-world patient access to biologic medicines only based on their availability on the national list of reimbursed medicines may fall short in CEE countries. As described in **Table 3**, some eligible CEE patients do not have timely access to the treatment, while the process behind the selection of treated patients remains untransparent and unpredictable, especially because informal payments or clientelism have longstanding tradition in some of the CEE countries (EC, 2013). Consequently, patients with similar clinical conditions may not have equitable access to the most appropriate treatment and their health perspectives may depend on their socio-economic status, geographical location or other social, financial, and organizational factors.

Hidden access barriers to reimbursed medicines are rarely published or even disclosed at public forums due to their political sensitivity. Therefore, countries with limited resources cannot directly learn from each other, and they need to develop an individualistic approach to ensure the sustainability of financing pharmaceuticals. This may explain the heterogeneity of reported access barriers across the five participant CEE countries.

While not all patient access barriers are relevant in each participant country (as shown in **Table 3**), some general conclusions can be drawn. First, access barriers exist everywhere, and were reported from all five countries. It is also quite likely that other CEE countries have also developed similar access barriers to improve the financial sustainability of high cost pharmaceuticals. Moreover, utilization data of biologics in RA suggests that some barriers, usually at smaller extent, may also exist in certain higher-income countries (Degli Esposti et al., 2019). Second, although budget control mechanisms influence all relevant stakeholders, compared to health care professionals and manufacturers, ultimately patients with limited negotiation power are the most vulnerable beneficiaries who suffer the most from these barriers. Third, the pattern of barriers is very “colorful” in each country, which confirm the creativity of policymakers in the CEE region to facilitate the sustainability of underfinanced health care system. However, some of the access problems may be related to an actual dysfunctioning of the health care system in CEE countries rather than to conscious cost-control mechanisms. Even in such cases, payers have limited incentives to overcome such dysfunctioning as they support the sustainability of health care financing. Whilst the uncontrolled use of high-cost pharmaceuticals cannot be supported, such control measures should be transparent and should not further increase access inequity among patients.

Patient access barriers listed in **Table 3** are either specific to CEE countries or even if they exist in higher-income EU member states, they have more impact on patients in CEE. For example, in several higher-income European countries, the prescription rights of biologics may also be limited to RA centers; however, the health care system is more decentralized and less restrictive for patients in rural areas.

The association of RA patient population and the economic status of countries is well documented in the scientific literature (Putrik et al., 2016a). In less affluent countries the higher DAS-28 scores could be partially explained by the lower utilization of bDMARDs, delay in diagnosis and access to rheumatological care (Putrik et al., 2016b).

Perception about the relevance and importance of access barriers may be different across stakeholder groups. For example, physicians or payers may advocate that continuation of biologic medicines should be subject to frequent monitoring of patients’ health status. While this may not be perceived as a barrier by patients living in the capital, it may represent an important access restriction and travel burden for elderly patients in rural areas. The example also highlights that combinations of hidden barriers (e.g., the availability of bDMARDs only in the centers, the need to frequently renew prescription even for good responders, mandatory interim diagnostics between two intravenous doses of bDMARDs for therapy continuation etc.) may have greater additive impact than isolated access barriers.

### Limitation

The primary objective of the study was to provide an exhaustive list of existing access barriers in a sample of countries from the CEE region, therefore even a single mention validated by the primary investigators was considered valuable. Due to the limited sample size responses could not be evaluated by statistical analyses. On the other hand, the research was intended to be qualitative, hence selection of the most important barriers and ranking among them was not in the study scope. Prioritization among access barriers in terms of their relative importance would require further and larger-scale research. For similar reasons, the study could not quantify the additive effects of multiple similar barriers. Finally, although authors made an attempt to reduce overlaps between the barriers, the possibility of double counting could not be fully excluded.

### Future Research

This exploratory research may be continued by (1) exploring the national context of the barriers, (2) determining the relative importance of the barriers including their interaction or mixed use (i.e., weighting or considering their additive effect), and (3) proposing specific policy actions to reduce the negative effects of hidden access barriers.

The implications of patient access barriers on drug utilization can be validated by quantitative studies using standard utilization metrics such as defined daily dose/1,000 inhabitants/day corrected for diseases prevalence. It is, however, difficult to conduct such research for TNF alpha inhibitors as they are used in multiple and not overlapping indications.

Further research is needed to quantify the implications of limited patient access to TNF alpha inhibitors—including no access, access only in advanced disease stages, or limited therapy duration—on the health status of RA patients and long-term treatment costs in CEE countries.

It should also be noted that some of the reported problems may have additional negative collateral effects in addition to limiting patient access to bDMARDs. For example, shortage of health care personnel or administrative burden to prescribe biologics can limit the capacity of overburdened physicians and nurses to participate in continuous medical education or to manage comorbidities of patients (Dougados et al., 2014), which may also have a negative impact on patients’ health. These factors may also incentivize migration of demotivated and underpaid health care professionals to higher-income countries (Győrffy et al., 2018; Domagala and Dubas-Jakobczvk, 2019). As the scope of our study was limited to explore patient access barriers to TNF alpha inhibitors, further research is needed to review these collateral effects.

## Conclusion

In many countries real-world patient access towards high cost pharmaceuticals has been solely estimated based on whether they are available on the list of reimbursed pharmaceuticals or not. So far, only limited empirical evidence has been published on the existence of transparent and especially on hidden patient access barriers implemented for high-cost biologics to improve the sustainability of health care financing. In order to initiate policy actions to reduce inequities in timely patient access, better evidence on access barriers is needed. To our best knowledge, this is the first study to generate scientific evidence on the access barriers to biologics in the CEE region relevant for any of the different major stakeholder groups in the example of RA by using a systematic approach. This research may be used as a prototype to reveal hidden access restrictions related to other pharmaceuticals in additional diseases. Once hidden barriers have been fully mapped, transparent, and predictable pharmaceutical policies, such as extended use of biosimilars (Inotai et al., 2017), should be implemented in CEE countries to improve equity in patient access without compromising financial sustainability of health care financing.

## Data Availability Statement

The datasets generated for this study will not be made publicly available to protect full anonymity of the respondents.

## Ethics Statement

The study protocol was approved by the Medical Research Council — Scientific and Ethical Committee in Hungary (number of ethical approval: 10220-2/2019/EKU), and research was performed in accordance with the ethical standards laid down in the 1964 Declaration of Helsinki. According to national legislation patient representatives were asked to sign a written informed consent form.

## Author Contributions

AI, DT, MN, and ZK contributed to the concept and design of the study. A-KK and AI performed the literature review. AI conducted the interviews in Hungary and coordinated the primary investigators. MK and JW conducted the interviews in Czech Republic, MN conducted the interviews in Poland, LL conducted the interviews in Romania and DT conducted the interviews in Slovakia. AI drafted the manuscript. A-KK, EK, and ZK revised the manuscript. All authors contributed to manuscript revision, read and approved the submitted version.

## Funding

The authors declare that this study received funding from Egis Pharmaceuticals PLC for the research and from the Biosimilar Medicines Group, a Medicines for Europe sector group, for the publication. The funders had the opportunity to review the manuscript before submission, however, were not involved in study design, or collection, analysis, interpretation of data or the decision to submit it for publication.

## Disclaimer

The authors summarized their independent professional opinion and take full responsibility for potential errors in the manuscript. The content of this paper, as well as the views and opinions expressed therein are those of the authors and not their affiliated organizations.

## Conflict of Interest

Authors AI and ZK were employed by the company Syreon Research Institute. Syreon Research Institute received financial support from Egis Pharmaceuticals PLC for the research and from the Biosimilar Medicines Group, a Medicines for Europe sector group, for the publication.

The remaining authors declare that the research was conducted in the absence of any commercial or financial relationships that could be construed as a potential conflict of interest.

## Abbreviations

bDMARDs, biologic disease modifying anti-rheumatic drugs; CEE, Central Eastern Europe; CZ, Czech Republic; EFPIA, European Federation of Pharmaceutical Industries and Associations: HU, Hungary; PL, Poland; RA, rheumatoid arthritis; RO, Romania; SK, Slovakia; TNF, tumor necrosis factor.
